# Integration of Alcohol Use Disorder Interventions in General Health Care Settings in Sub-Saharan Africa: A Scoping Review

**DOI:** 10.3389/fpsyt.2022.822791

**Published:** 2022-03-15

**Authors:** Dorothy Mushi, Joel M. Francis, Candida Moshiro, Charlotte Hanlon, Solomon Teferra

**Affiliations:** ^1^Department of Psychiatry, WHO Collaborating Centre for Mental Health Research and Capacity-Building, School of Medicine, College of Health Sciences, Addis Ababa University, Addis Ababa, Ethiopia; ^2^Centre for Innovative Drug Development and Therapeutics Trial for Africa, College of Health Sciences, Addis Ababa University, Addis Ababa, Ethiopia; ^3^Department of Psychiatry and Mental Health, Muhimbili University of Health and Allied Science, Dar es Salaam, Tanzania; ^4^Department of Family Medicine and Primary Care, Faculty of Health Sciences, Witwatersrand University, Johannesburg, South Africa; ^5^Department of Epidemiology and Biostatistics, Muhimbili University of Health and Allied Science, Dar es Salaam, Tanzania; ^6^Centre for Global Mental Health, Health Service and Population Research Department and WHO Collaborating Centre for Mental Health and Training, Institute of Psychiatry, Psychology, and Neuroscience, King's College University, London, United Kingdom

**Keywords:** alcohol use disorder intervention, integration of intervention for alcohol use disorder, mental health plan, screening, brief intervention, sub-Saharan Africa, general health care

## Abstract

**Introduction:**

Alcohol use disorder (AUD) is among the leading cause of morbidity and mortality in sub-Saharan Africa. Despite this, AUD is often not detected in health care settings, which contributes to a wide treatment gap. Integrating services for mental, neurological, and substance use disorders in general health care settings is among the recommended strategies to narrow this treatment gap. This scoping review aimed to map the available evidence on integration of AUD interventions in general health care settings in sub–Saharan Africa.

**Methods:**

We searched four databases (PubMed, PsycINFO, CINAHL, and Africa Wide Information) for publications up to December 2020. The search strategy focused on terms for alcohol use, alcohol interventions, and sub-Saharan African countries. Studies that reported AUD interventions in general health care settings in sub–Saharan Africa were eligible for inclusion. Over 3,817 potentially eligible articles were identified. After the removal of duplicates and screening of abstracts, 56 articles were included for full article review. Of these, 24 papers reporting on 22 studies were eligible and included in a narrative review.

**Results:**

Of the 24 eligible articles, 19 (80%) described AUD interventions that were being delivered in general health care settings, 3 (12%) described plans or programs for integrating AUD interventions at different levels of care, including in health facilities, and 2 (8%) studies reported on AUD interventions integrated into general health care settings.

**Conclusions:**

This review shows that there is limited evidence on the integration of AUD interventions in health care settings in sub-Saharan Africa. There is an urgent need for studies that report systematically on the development, adaptation, implementation, and evaluation of integrated AUD interventions in health care settings in sub-Saharan Africa.

## Introduction

Alcohol Use Disorder (AUD) is defined as a cluster of cognitive, behavioral, and physiological symptoms indicating that the individual continues using alcohol despite significant alcohol-related problems (1). AUD accounts for about 5.1% of all disability-adjusted life years (DALYs) and 5.3% of all deaths globally (2). AUD is also a risk factor for many diseases, injuries, and social issues including child neglect and violence (2). In addition, AUD negatively impacts the economic wellbeing of both individuals and society at large (2).

Studies conducted in sub-Saharan Africa (SSA) have shown that approximately one in five people attending health care facilities meet the criteria for AUD (3–7). AUD in the African setting is associated with injuries (5, 8), physical and mental health problems (9), as well as direct and indirect adverse effects on HIV disease progression (6), barriers to seeking professional help, stigma, and a low tendency to seek help (9, 10). Even though the magnitude and factors associated with AUD are substantial, AUD appears to be rarely detected by health care providers (11) leading to a wide treatment gap (9, 11–13). This gap is particularly marked in low-income and lower-middle-income countries (9, 11, 12).

The World Health Organization (WHO) mental health Gap Action Program (mhGAP) intervention guidelines support the integration of services for people with mental health, substance use, and neurological disorders into general and primary health care as a means of narrowing the treatment gap (14–16). In SSA, interventions for AUD have been reported in various general health care settings (3, 17–27), including services for people with HIV or other sexually transmitted diseases (9, 10, 13–16, 18, 21), antenatal and postnatal care (19, 27–29), care for people with tuberculosis (30, 31), inpatient and outpatient care (20, 26, 32–35). Some of these studies have further reported on implementation outcomes (e.g., feasibility or acceptability) of interventions for AUD in general health care settings (7, 36–38). However, evidence on the integration of AUD interventions into general health care settings has not been synthesized.

In response to this gap, in this scoping review, we mapped the available evidence on the integration of interventions for people with AUD attending general health care facilities in SSA.

## Methods

### Design

We conducted a scoping review of published articles that described or evaluated interventions, service models, plans, or programs for integrating care for people with AUD in general health care facilities in SSA.

A scoping review can be undertaken to map the key concepts underpinning a research area as well as to clarify working definitions, and/or the conceptual boundaries of a topic (39). This study was guided by Arksey and O'Malley's (39) scoping review methodological framework. Furthermore, the study team followed the PRISMA-ScR (Preferred Reporting Items for Systematic reviews and Meta-Analyses extension for Scoping Reviews) checklist and explanation (40). Based on the WHO technical report on the integration of health services, we have defined integration as a range of services/interventions for alcohol use disorder integrated into the work of general health care workers in general health care settings (41).

This study is part of formative work to adapt and pilot an integrated intervention model for people with AUD in primary healthcare in rural Tanzania (42).

### Search Strategies

We reviewed published literature on interventions for AUD integrated into general health care settings in SSA. The search was conducted using the following electronic databases: PubMed, PsychINFO, CINAHL, and Africa Wide Information. We included studies published from the inception of databases to December 2020 (Supplementary Material). The rationale for this was that no previous review on integration of interventions for people with AUD attending general health care facilities in SSA had been conducted. We also anticipated that older studies would still have potential relevance. All types of study designs were included.

Two reviewers (DM and JF) independently screened the titles and abstracts of citations identified through the search strategy, and thereafter full articles, to select studies that met inclusion criteria. Disagreements between the screeners were resolved by a joint discussion, without the need for the involvement of senior co-investigators.

### Inclusion Criteria

(i) We included studies that reported on any interventions for AUD conducted among people attending general health care facilities in SSA.
(ii) Studies with any methodology (quantitative, qualitative, mixed) were included.

### Exclusion Criteria

(i) Studies that reported on interventions for AUD in other settings, for example, in specialist settings were excluded.
(ii) Systematic reviews and meta-analyses studies were excluded, but the individual studies included within these papers were assessed for eligibility.

### Extraction of the Data

A pre-tested data extraction form was used to extract the information needed for this review. A pre-test was conducted with five articles to assess if all the required information would be captured. The following information was extracted:

(i) author, (ii) country in which the study was conducted, (iii) year the study was conducted, (iv) year of publication, (v) study population and type of health care service, (vi) type of AUD interventions, (vii) primary outcome/s, and (viii) integration status of AUD intervention.

## Results

### Identified Papers

**Figure 1:**
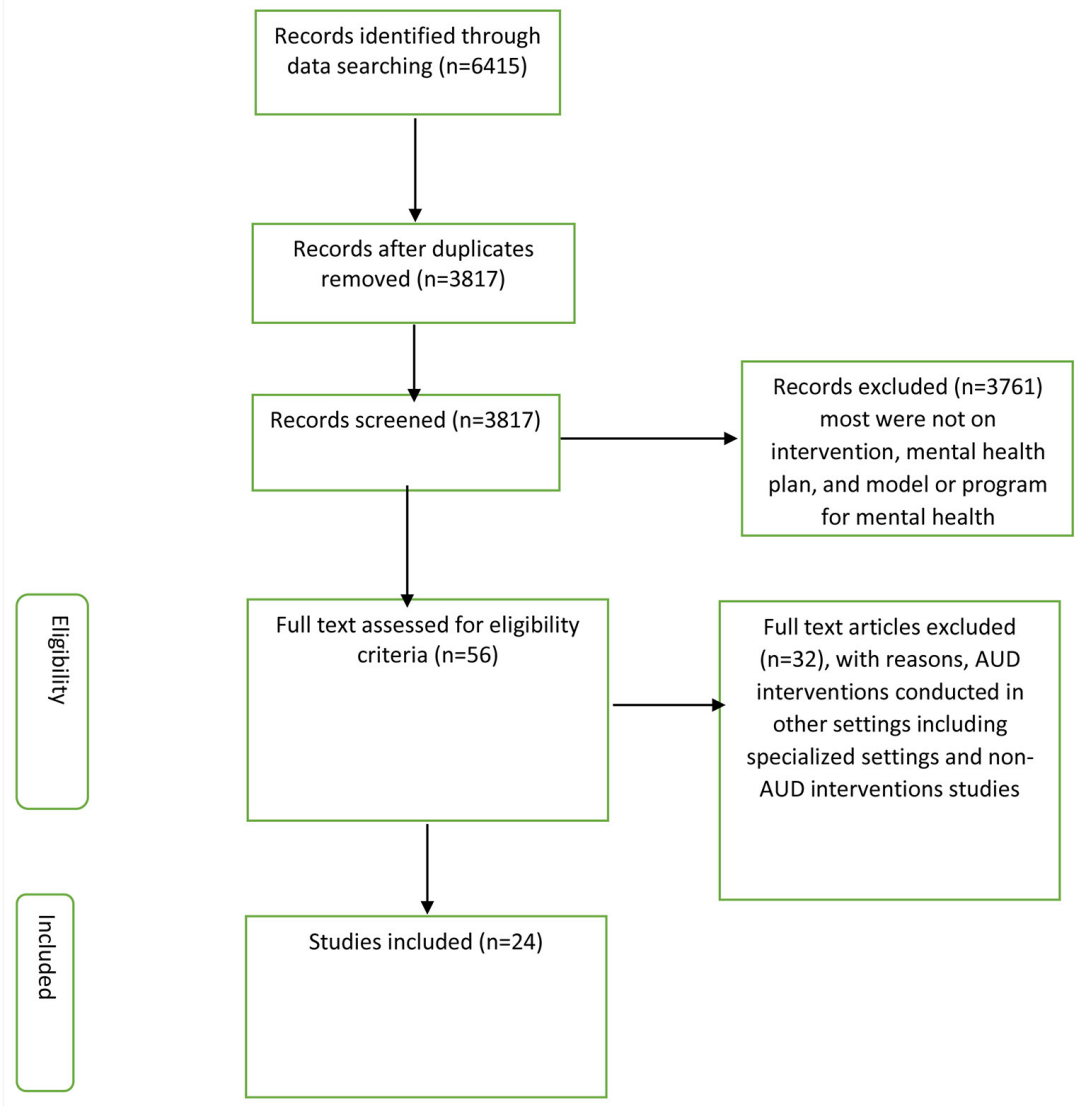
Flow diagram of the identified citation and included papers.

In this review, 24 articles were eligible for the synthesis of information. Figure 1 presents the overall process of searching the potential articles included in this review. The review yielded 6,415 citations, out of which 2,598 (40%) were duplicates. We screened titles and abstracts of 3,817 citations and identified 56 citations for full article review. Of these, we excluded 32 articles that did not report on AUD interventions or reported on AUD interventions from settings other than general health care.

We present the review findings as follows:

(i) the underlying study design, (ii) targeted population and type of health care service, (iii) region of sub-Saharan Africa where the study took place, (iv) publication year, (v) primary outcome, and (vi) AUD intervention.

### Study Designs of the Included Studies

Most of the studies were randomized controlled trials (*n* = 15), followed by mixed-method study designs (*n* = 7), cohort study design (*n* = 1), and cross-sectional survey (*n* = 1).

### Health Care Service and Targeted Population

Most studies were of AUD interventions integrated within HIV care or services for other sexually transmitted diseases (*n* = 10), or in general clinical services (*n* = 10), with the remainder conducted in the context of emergency services (*n* = 3) and tuberculosis care/clinics (*n* = 1).

### Sub-Saharan Africa Regions Where Interventions Were Implemented

Most of the studies were implemented in the southern African region (*n* = 12) and eastern region (*n* = 10), with two studies conducted in the central region, and one in the western region.

### Publication Year of the Interventions

Most studies were published from the years 2010 to 2020 (*n* = 20), while three studies were reported from the years 2000 to 2010. Only one study was published before the year 2000.

### Integrated Interventions for AUD in General Health Care Settings

We found several studies describing AUD interventions delivered in general health settings in SSA. However, for most of these studies, we could not be certain if those interventions were integrated into these services. In addition to that, we also found studies that reported frameworks and plans for integrated interventions for the mental health services that also included AUD care as one component. Based on this observation we have reported our results in three categories: (i) studies that described interventions for AUD, (ii) studies that described plans or frameworks for integrated AUD interventions, and (iii) studies that reported the integration status of AUD interventions in general health care settings.

### Interventions for AUD in General Health Care Settings

Table 1 describes the interventions for AUD that have been applied in general health care settings in SSA, which included: (i) interventions which were based on motivational interviewing techniques (*n* = 15), (ii) interventions focused on identification of alcohol use (*n* = 8), (iii) interventions which applied cognitive behavior therapy (*n* = 3), (iv) interventions that focused on risk/behavioral reduction counseling (*n* = 1), and (v) interventions that applied problem solving skills (*n* = 1).

**Table 1:** Description of interventions for alcohol use disorder in Sub Saharan Africa general health care settings.

	**References**	**Publication**	**Study year**	**Study design**	**Country**	**Targeted population and type of health care service**	**Alcohol intervention**	**Primary outcome**
**1**	Ward et al. (43)	2015	Not reported	Randomized control trial	South Africa	Patient in community health care	Screening and brief intervention	Reduced alcohol use, aggression, and HIV risk behavior
**2**	Myers B et al. (26)	2012	Not reported	Mixed method	South Africa	Patient in emergency services	Screening and brief motivation intervention	Feasibility on conducting peer led Screening
**3**	Sorsdahl et al. (44)	2014	Not reported	Mixed method	South Africa	Patient and peer counselor in emergency service	Blended motivation interviewing and problem-solving intervention	Optimal delivery of blended MI and PST
**4**	Madhombiro et al. (45)	2019	2016	Randomized control trial	Zimbabwe	Patient and health care providers in HIV care	Brief motivation interviewing and cognitive behavioral therapy (MI/CBT)	Feasibility on delivery of brief MI/CBT
**5**	Tang et al. (46)	2019	Not reported	Mixed method	Namibia	Patient in HIV care	Screening and brief intervention	Implementation and evaluation of SBI
**6**	Mertens et al. (47)	2014	Between March and November 2008	Randomized control trial	South Africa	Patient and health care providers in primary health care	Screening and brief motivation intervention	Optimal delivery of Brief motivational interventional
**7**	Papas et al. (48)	2010	Not reported	Randomized control trial	Kenya	Patient in HIV care and paraprofessional providers	Cognitive behavioral therapy	Optimal delivery of CBT
**8**	Huis In ‘t Veld et al. (49)	2019	2012 to 2013	Randomized control trial	South Africa	Patient in HIV care	Screening and brief motivation intervention	Reduced alcohol use
**9**	Papas et al. (50)	2011	Not reported	Randomized control trial	Kenya	Patient in HIV care	Cognitive behavioral therapy	Reduced alcohol use
**10**	Hahn et al. (24)	2014	2008 to 2011	Randomized control trial	Uganda	Patient in HIV care	HIV counseling and testing	Reduced alcohol use
**11**	Multicountry study (51)	1996	Not reported	Randomized control study	Kenya and Zimbabwe	Patient in general health care	Brief motivation intervention	Reduced alcohol use
**12**	Kalichman et al. (25)	2007	2005 and 2006	Randomized control trial	South Africa	Patient in sexually transmitted clinic	Brief alcohol counseling model	Reduced alcohol use
**13**	Ramarumo et al. (35)	2016	Not reported	Randomized control trial	South Africa	Patient in outpatient care	Brief counseling session	Reduced alcohol use
**14**	Wandera et al. (21)	2017	October 2012 and May 2013	Randomized control trial	Uganda	Patient in HIV care	Standardized information and brief counseling based on Motivation intervention	Reduced alcohol use
**15**	Peltzer et al. (52)	2013	April to October 2011	Randomized control trial	South Africa	Patient in a tuberculosis clinic	Brief intervention	Reduced alcohol use
**16**	Emenyonu et al. (53)	2017	June 2013	Randomized control trial	Uganda	Patient in HIV care	Alcohol use assessment	Reduced alcohol use
**17**	Clair et al. (54)	2019	October 2014 and March 2015	Randomized control trial	Kenya	Health care providers and patients in primary health care	Motivation interviewing using the mobile phone, or in-person	Reduced alcohol use
**18**	Harder et al. (55)	2019	Between October 2014 and March 2015	Randomized control trial	Kenya	Patient in general health care	Mobile motivation intervention	Effectiveness of motivational interviewing using mobile phone
**19**	Van der Westhuizen et al. (56)	2019	August 2016 to July 2017	Mixed method	South Africa	Patient and peer counselor in emergency service	Screening, Brief Intervention, Referral to Treatment (SBIRT)	Evaluating feasibility, acceptability, appropriateness, and adoption of the task-shared SBIRT program

### Programs for Integrated AUD Interventions

This included district mental health plans (*n* = 2) and a program intervention guide (*n* = 1) for integrated mental health services that included AUD (Table 2). The implementation outcomes [such as acceptability and feasibility (58)] for the integrated mental health services that included AUD were also reported in these studies. Strategies that facilitated the implementation of AUD care within integrated mental health services at different levels of care (community, general health facility, health system organization) were also reported. The following implementation strategies were reported: (i) engaging mental health stakeholders from inception to enhance their ownership and commitment, (ii) conducting sensitization workshops (37, 38), (iii) training clinical staff (36–38), (iv) supervising decision support and supporting staff well-being (3–37, 59), and leveraging available resources and existing systems (36–38). In addition, these studies identified individual and structural bottlenecks for implementing integrated mental health services including AUD (36, 38).

**Table 2:** Description of integrated interventions, mental health plan and program for alcohol use disorder in Sub Saharan Africa general health care settings.

	**References**	**Publication year**	**Study year**	**Study design**	**Country**	**Targeted population**	**Alcohol intervention**	**Primary outcome**
**1**	Peltzer et al. (7)	2008	Not reported	Cross-sectional	South Africa	Patient in a general clinic	Screening and brief motivation intervention	Implementation of screening and brief motivational interventional
**2**	Cagle et al. (57)	2017	Not reported	Retrospective cohort study	Kenya	Patient in HIV care	Standardized questionnaire	Alcohol use detection
**3**	^*^Gureje et al. (36)	2015	Not reported	Mixed methods	Nigeria	Health care providers	Contextualize World Health Organization Mental Health Gap Action Program Intervention Guide (mhGAP-IG),	Implementation of mhGAP-IG
**4**	^*^Fekadu et al. (37)	2018	Not reported	Mixed methods	Ethiopia	Mental health stakeholders	Mental health care plan (MHCP)	Development of the MHCP
**5**	^*^Petersen et al. (38)	2018	Not reported	Mixed methods	South Africa	Mental health stakeholders	District mental health care plan	Development of the MHCP

* *Studies that provide framework/guidance on mental health plans and programs for mental health services include AUD*.

### Integrated Interventions for AUD in General Health Care Settings

This included routine screening or inquiring about alcohol use disorder (*n* = 1), and screening and brief intervention (SBI) for AUD (*n* = 1) (Table 2). These studies aimed to assess routine screening for alcohol use for people living with HIV during enrollment in the HIV clinic (57) and implementation of an alcohol screening and brief intervention in 18 primary health care services (7). The study found nine clinics had good and nine had poor SBI implementation. To improve routine implementation of SBI, the study recommended that more attention needs to be paid to training, clinic organization, and addressing the attitudes of health care providers.

## Discussion

In this review, we found only two studies that described and reported on the integration status of AUD interventions in general health care settings in SSA (7, 57). Importantly, we identified several studies detailing the mental health plans and programs based on integrated interventions for mental health services that include AUD (36–38). Our review findings are in keeping with previous reviews that reported a paucity of interventions to address problematic alcohol use in SSA (60, 61). Our findings reflect the low priority accorded to expanding access to care for AUD indicated in the World Health Organization (WHO) report on alcohol and health in low and middle-income countries (2). In that WHO report, treatment coverage for AUD is low and national alcohol policies are scarce in SSA.

Nevertheless, the identified studies indicated that it is possible to implement integrated mental health services, including AUD care, in general, health care settings in SSA. This is similar to findings from a brief review of integrating interventions for AUD into clinical practice in high-income settings (62). These studies demonstrated that AUD care, as a component of integrated mental health care, can achieve a positive and significant impact on clinical outcomes for people with AUD (36, 38). Additionally, these studies also identified various strategies that may facilitate the implementation of AUD components of an integrated mental health care program. These strategies included methods to improve detection, support decision making, improve staff wellbeing, and ensure proper program management and supportive supervision (36–38). These findings align with a previous review on strategies to facilitate integrated care for people with alcohol problems (63).

Individual and structural bottlenecks to the implementation of integrated interventions for mental health services including AUD were also identified. These findings are in keeping with results from a review of barriers and facilitators to implementing screening and brief intervention for alcohol misuse in high-income country settings (64).

This scoping review had some limitations. We were not able to document policy level examples of integration of AUD, and we did not search the gray literature where program evaluations may be available. The reasons for omitting a gray literature search was largely due to feasibility, but also reflected our concerns that some gray literature may not be published online or the uniform resource locator (URL)/website may not be stable, older documents may not be archived, and format and citation information could be inconsistent.

It is likely that other examples of integration of AUD interventions in general health care exist but have not been reported and, therefore, could not be detected by this scoping review.

## Conclusions

This review has highlighted the paucity of studies on integrated interventions for AUD in general health care settings in sub-Saharan Africa. There is an urgent need for studies that report systematically on the development, or adaptation, implementation, and evaluation of integrated AUD interventions in health care settings in sub- Saharan Africa.

## Data Availability Statement

The original contributions presented in the study are included in the article/Supplementary Material, further inquiries can be directed to the corresponding author.

## Author Contributions

DM developed the study design with contribution from JF, CH, CM, and ST. DM and JF carried out the article's systematic searching, screening, and eligibility checking. DM performed data analysis with contribution from JF. DM wrote the manuscript draft. All authors took part in the interpretation of the data, reviewed the draft, provided important intellectual materials, and agreed on the final draft.

## Funding

DM acknowledges support from the Centre for Innovative Drug Development and Therapeutics Trial for Africa (CDT-Africa), a World Bank Africa Centre of excellence at Addis Ababa University as part of her training fellowship.

## Author Disclaimer

The opinions in this paper are those of the authors and do not reflect the views of CDT-Africa.

## Conflict of Interest

The authors declare that the research was conducted in the absence of any commercial or financial relationships that could be construed as a potential conflict of interest.

## Publisher's Note

All claims expressed in this article are solely those of the authors and do not necessarily represent those of their affiliated organizations, or those of the publisher, the editors and the reviewers. Any product that may be evaluated in this article, or claim that may be made by its manufacturer, is not guaranteed or endorsed by the publisher.
