# Supplementary material for: Integration of Alcohol Use Disorder Interventions in General Health Care Settings in Sub-Saharan Africa: A Scoping Review
Source: Front Psychiatry. 2022 Mar 15;13:822791. doi: 10.3389/fpsyt.2022.822791 (PMC8964495; doi:10.3389/fpsyt.2022.822791)
Supplement: Supplementary file 1 [file Table_1.DOCX]

**SEARCH TERMS**

Key terms… alcohol, interventions for alcohol use disorders, sub-Saharan Africa

**ALCOHOL**

**Related terms** : -Alcohol OR “Alcohol-Induced Disorders” OR “Alcohol-Related Disorders” OR “Alcohol Drinking” OR “Underage Drinking” OR ‘’Alcohol Withdrawal Delirium” OR Alcoholism OR “alcohol abuse” OR “alcohol intoxication” OR ‘’alcohol use disorder” OR ‘’hazardous drinking” OR ‘’harmful drinking” OR “alcohol dependence” OR ‘’drinking behaviour” OR ‘’Alcohol Consumption” OR “Alcohol misuse” OR ‘’Problematic alcohol use”

AND

**2) Interventions for AUD**

Related terms "Early Medical Intervention" OR "Early Intervention’’ OR “ brief intervention” OR “alcohol brief intervention” OR “alcohol reduction” OR “alcohol screening” OR “alcohol therapy” OR “alcohol treatment” OR “harm reduction” OR “controlled drinking” OR “ brief counselling “OR “physician- based intervention” OR “ general practioner based intervention” OR “general practice intervention” OR “secondary intervention” OR “ secondary prevention” OR “general practitioner’s advice” OR “brief physician-delivered counseling” OR” brief nurse-delivered counseling “ OR screening OR Diagnosis OR detection OR " detection of alcohol use “ OR ‘’ identification of alcohol use’’ OR ‘’assessment of alcohol’’ OR "Early Diagnosis" OR ‘’Alcohol management’’ OR “recognition of alcohol use” OR ‘’Psychosocial intervention’’ OR ‘’Psychosocial care’’ OR ‘’ psychological treatment’’ OR ‘’Pharmacological intervention’’ OR’ ‘’Case management intervention’’ OR ‘’Community intervention’’ OR Detoxification OR ‘’Drug detoxication’’ OR **‘’**Community outreach’’ OR ‘’community services’’

AND

**3) Sub Saharan Africa**

"Africa South of the Sahara" OR ‘’Sub-Saharan Africa’’ OR ‘’Africa, Sub-Saharan’’ OR  **‘’**Africa South of the Sahara’’ OR ‘’Africa central’’ OR ‘’Africa, Eastern’’ OR ‘’Africa, Southern’’ OR ‘’Africa, Western’’ OR ‘’Less developed’’ OR ‘’less developed nation’’ OR ‘’third world nation’’ OR ‘’third world country’’ OR ‘’under developed nation’’ OR ‘’under developed country’’ OR ‘’middle income country’’ OR ‘’middle income nation’’ OR ‘’low income country’’ OR ‘’low income nation’’ OR ‘’poor country’’ OR ‘’poor nation’’ OR Angola OR Benin OR ‘’Burkina Faso’’ OR Burundi OR ‘’Cabo Verde ’’ OR ‘’Cape Verde’’ OR Cameroon OR ‘’Central African’’ OR Chad OR Comoros OR Congo OR ‘’Costa Rica’’ OR ‘’Cote d'Ivoire’’ OR ‘’Ivory Coast’’ OR Djibouti OR Eritrea OR Ethiopia OR Gabon OR Gambia OR Ghana OR Guinea OR Kenya OR Lesotho OR Liberia OR Libya OR Madagascar OR Malawi OR Mali OR Mauritania OR Mozambique OR Namibia OR Niger OR Rwanda OR Samoa OR ‘’Sao Tome and Principe’’ OR Senegal OR ‘’Sierra Leone’’ OR Somalia OR South Africa OR Sudan OR Swaziland OR Tanzania OR Togo OR Tunisia OR Uganda OR Zambia OR Zimbabwe
